# Towards standardized antifungal susceptibility testing for *Sporothrix brasiliensis*

**DOI:** 10.1093/jac/dkag286

**Published:** 2026-08-19

**Authors:** Bram Spruijtenburg, Carolina Melchior Do Prado, Mariana Rodrigues Trápaga, Dallas J Smith, Philippe J Dufresne, Jacques F Meis, Larissa Fernandes, Maria Sueli Felipe, Daniel R Matute, Theun De Groot, Amanda Ribeiro Dos Santos, Regielly Caroline Raimundo Cognialli, Nathan P Wiederhold, Vânia A Vicente, Flavio de Queiroz-Telles, Melissa Orzechowski Xavier, Marcus de Melo Teixeira, Eelco F J Meijer

**Affiliations:** Department of Medical Microbiology, Radboudumc, Nijmegen, The Netherlands; Radboudumc-CWZ Center of Expertise for Mycology, Nijmegen, The Netherlands; Department of Medical Microbiology and Immunology, Canisius-Wilhelmina Hospital (CWZ)/Dicoon, Nijmegen, The Netherlands; Radboudumc-CWZ Center of Expertise for Mycology, Nijmegen, The Netherlands; Department of Medical Microbiology and Immunology, Canisius-Wilhelmina Hospital (CWZ)/Dicoon, Nijmegen, The Netherlands; Post-graduation Program in Health Science, Faculty of Medicine (FAMED), Federal University of Rio Grande (FURG), Rio Grande, Brazil; Mycology Laboratory, FAMED-FURG, Rio Grande, Brazil; Mycotic Diseases Branch, Centers for Disease Control and Prevention, Atlanta, Georgia, USA; Laboratoire de Santé Publique du Québec, Institut National de Santé Publique du Québec, Sainte-Anne-de-Bellevue, Québec, Canada; Department of Medical Microbiology, Radboudumc, Nijmegen, The Netherlands; Radboudumc-CWZ Center of Expertise for Mycology, Nijmegen, The Netherlands; Institute of Translational Research, Cologne Excellence Cluster on Cellular Stress Responses in Aging-Associated Diseases (CECAD) and Excellence Center for Medical Mycology, University of Cologne, Cologne, Germany; Faculty of Ceilândia, University of Brasília, Brazil; Postgraduate Program in Genomic Sciences and Biotechnology, Universidade Católica de Brasília, Brasília, Brazil; Universidade de Brasília, Brasília, Brazil; Department of Biology, University of North Carolina at Chapel Hill, Chapel Hill, NC, USA; Radboudumc-CWZ Center of Expertise for Mycology, Nijmegen, The Netherlands; Department of Medical Microbiology and Immunology, Canisius-Wilhelmina Hospital (CWZ)/Dicoon, Nijmegen, The Netherlands; Department of Clinical and Toxicological Analyses, School of Pharmaceutical Sciences, São Paulo University (USP), São Paulo, Brazil; Postgraduate Program in Microbiology, Parasitology and Pathology, Biological Sciences, Department of Basic Pathology, Federal University of Paraná, Curitiba, Brazil; Hospital de Clínicas, Federal University of Paraná, Curitiba, Brazil; University of Texas Health Science Center at San Antonio, San Antonio, TX, USA; Postgraduate Program in Microbiology, Parasitology and Pathology, Biological Sciences, Department of Basic Pathology, Federal University of Paraná, Curitiba, Brazil; Microbiology Collections Center of Paraná Network-Taxonline (CMRP/Taxonline), Department of Basic Pathology, Federal University of Paraná, Curitiba, Brazil; Department of Public Health, Hospital de Clínicas, Federal University of Paraná, Curitiba, Brazil; Post-graduation Program in Health Science, Faculty of Medicine (FAMED), Federal University of Rio Grande (FURG), Rio Grande, Brazil; Mycology Laboratory, FAMED-FURG, Rio Grande, Brazil; School of Medicine, University of Brasilia, Brasilia, Brazil; Department of Medical Microbiology, Radboudumc, Nijmegen, The Netherlands; Radboudumc-CWZ Center of Expertise for Mycology, Nijmegen, The Netherlands; Department of Medical Microbiology and Immunology, Canisius-Wilhelmina Hospital (CWZ)/Dicoon, Nijmegen, The Netherlands

## Abstract

Sporotrichosis caused by *Sporothrix brasiliensis* is increasingly reported in South America. Infections in humans are mostly due to zoonotic transmission by cats. For both hosts, itraconazole is the drug of choice despite long treatment duration. Resistance surveillance is warranted, but protocols for antifungal susceptibility testing (AFST) are not always harmonized between laboratories largely due to *Sporothrix* species being thermally dimorphic and the absence of well-defined consensus methodologies to address how to deal with yeast/mould phases.

To standardize susceptibility testing methodology, we propose to modify the current Clinical Laboratory and Standards Institute (CLSI) M38 broth microdilution methodology with *S. brasiliensis* culture for 7 days at 30°C (rather than 35°C) on potato dextrose agar (PDA), followed by microscopic confirmation of pure mycelial phase. Incubation temperature would also be modified to 30°C for 72 hours. By following this standardized method for at least itraconazole, terbinafine and amphotericin B, *S. brasiliensis* non-wild-type isolates and correlations with clinical outcomes can be properly investigated. The proposed method is a consensus-oriented interim approach rather than a fully validated standard.

Sporotrichosis is among the most common implantation mycoses with cases reported from all inhabited continents.^1^ While there are >60 described *Sporothrix* species, the *S. schenckii* complex encompassing *S. schenckii sensu stricto*, *S. brasiliensis* and *S. globosa,* is clinically the most relevant.^1,2^ Transmission occurs through sapronotic and zoonotic routes, while it may also occur via fomites.^3^ Notably, there are uncontrolled feline outbreaks in South America and to a lesser extent in Southeast Asia caused by *S. brasiliensis* and *S. schenckii*, respectively.^3,4^ Containment of this epidemic is further hindered by multiple independent emergences of virulent lineages, dissemination through fomites, pet movement and reports of reduced azole susceptibility *in vitro*, particularly to itraconazole, the first-line therapy in both humans and animals.^5,6^ This highlights the need for novel therapeutic strategies and more optimized treatment, especially after treatment failure or with refractory cases.

AFST is performed to assess the *in vitro* activity of antifungal agents to guide treatment. Despite standardized protocols for yeast and moulds, *Sporothrix* species pose a challenge in the laboratory as they are thermally dimorphic.^1^ At 25–30°C, the fungus displays the mycelial form while at 35°C it transforms to the yeast phase. Standard mycelial AFST protocols such as CLSI M38 are all performed at 35°C although the mould form is prescribed, this induces transition towards the yeast phase during incubation.^1,7^ While yeast-phase AFST is commonly conducted at 35°C using short incubation periods, these conditions frequently fail to promote complete dimorphic transition due to the slow growth of *Sporothrix* spp.^8^ Susceptibility testing might therefore be conducted on mixed or transitional populations composed of conidia, hyphal elements, yeast-like cells and intermediate morphotypes. As such, numerous reports on *Sporothrix* species mention modifications to standard AFST protocols. Alarmingly, most of these studies neither provide sufficient methodological detail nor confirm the morphological phase of the inoculum before and after AFST.^6,8^ This leads to inter-laboratory variation as the *in vitro* susceptibility to antifungals differs between the yeast and mycelial phases.^5,9^ This clearly raises the question of which adaptations to existing reference methods are required to accurately perform AFST of *Sporothrix* species. In this article, we discuss recent literature to propose the best method to determine the antifungal susceptibility of *Sporothrix brasiliensis*. We did not find any AFST studies of *S. schenckii* or *S. globosa* in which the susceptibility of mycelial versus yeast phase, as confirmed by microscopy, was compared. Owing to the increasing number of *S. brasiliensis* cases and potential reports of antifungal resistance, the current article focusses on this species only. Extrapolation of proposed AFST modifications in this article to other *Sporothrix* species should be made with caution due to their different growth characteristics, with differences even more pronounced for *S. globosa*.^9^

Microbroth dilution is the current reference method for AFST, with protocols by the CLSI and the EUCAST guidelines. Given that in-house preparation of microbroth plates is a time-consuming process, commercial plates based on CLSI methods, such as Sensititre Yeast One or MICRONAUT-AM, are increasingly incorporated in routine laboratory practices.^10^ For *Sporothrix*, the CLSI method is mostly used with only a few reports of AFST by EUCAST protocols.^11^ Next to microbroth dilution, drug gradient strips and disc diffusion are alternative methods that are less used.^12^ Although most investigations claim testing in either the yeast or mycelial phase, this is often not supported by microscopic confirmation.^13^

Antifungal susceptibility profiles of *S. brasiliensis* isolates were recently determined according to CLSI guidelines for both morphological phases, confirmed with microscopy before and after inoculation.^13^ For the mycelial phase, isolates were incubated at 30°C for a week on PDA, a nutrient poor medium promoting sporulation, followed by 72 hours at 30°C in microtitre plates with conventional RPMI-1640 medium. To achieve full transition to the yeast phase, a 1-week culture, followed by another week of culture was required, both at 35°C on brain–heart infusion agar, a nutrient rich medium. The inoculum was incubated for 5 days in RPMI-1640 at 35°C. Comparing antifungal susceptibility between both phases on a set of 108 genetically related isolates, the yeast phase demonstrated lower minimum inhibitory concentrations (MICs) for four different azoles and amphotericin B with often a broader range of MICs. Depending on the drug tested, MIC values of the same isolate showed up to eight 2-fold dilutions lower MIC values in the yeast phase when compared with the mycelial phase. While only wild-type isolates were found following this strict AFST methodology, a subsequent susceptibility study using the same protocol for the mycelial phase on a set of 450 *S. brasiliensis* isolates with more genetic variability found a high rate of itraconazole non-wild-type isolates, resulting in a bimodal MIC distribution. Similar results were found for a selection of isolates using the yeast-phase AFST protocol. Finally, another study also found a bimodal distribution for itraconazole, although details regarding the AFST protocol were lacking.^6,14^ Altogether, both phases are suitable to separate wild-type and non-wild-type isolates, although the yeast phase takes almost twice as long due to the time required for full transition to yeast phase and the subsequent inoculum incubation .

Despite the yeast phase being recognized as the pathogenic infecting form in the host and thereby clinically more relevant than the mycelial phase, we recommend AFST in the mycelial phase only, confirmed with microscopy before testing. This is mainly because of the shorter turn-around time, which is important to guide patient treatment.^15^ In addition, reduced hands-on laboratory time and incubation on the relatively inexpensive PDA medium make this approach more easily implementable in resource-limited settings. Regarding inoculum preparation, the outlined methodology for *S. schenckii* is sufficient although cell counting can be used for confirmation of the cfu. Furthermore, to maintain the mycelial phase we recommend incubating the microtitre plate at 30°C, thereby deviating from the CLSI M38 guideline, which states 35°C. We discourage the use of media other than PDA before inoculum incubation to obtain the pure mycelial phase, but if done so, microscopy is essential for confirmation. Conventional sodium hydroxide or lactophenol cotton blue wet mount slides are sufficient to properly assess the morphological phase (Figure 1).

**Figure 1.**
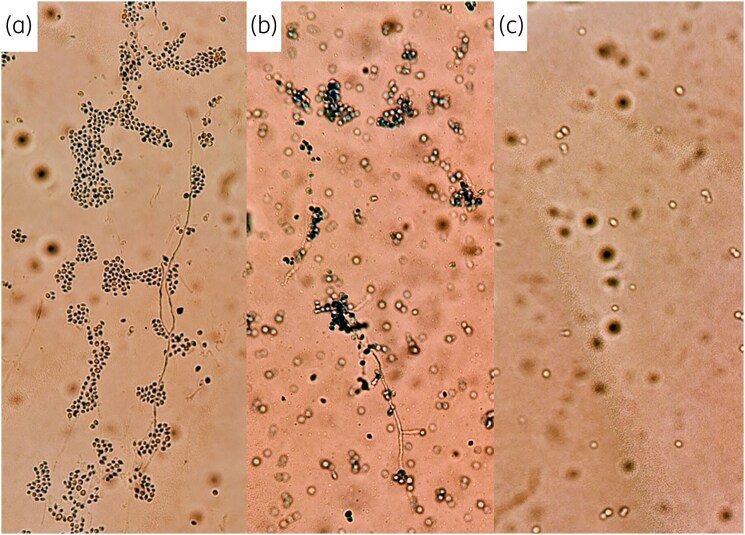
Microscopic morphology of *S. brasiliensis* under ×400 magnification stained with lactophenol cotton blue, with pure mycelial phase (a), a mix of mycelial and yeast (b) and pure yeast phase (c).

As MICs differ between the mycelial and yeast phase, results of previous studies cannot reliably be compared without morphology confirmation. When the mycelial phase is tested, reliable comparisons can be made between laboratories without elevated MICs, meaning MICs above the epidemiological cut-off value, being overlooked. Importantly, this allows assessment of antifungal non-wild-type rates between regions. Given that breakpoints are lacking for *S. brasiliensis*, multicentre studies need to be performed to correlate clinical outcome with *in vitro* susceptibility, as MICs are not predictive of clinical failure without defined breakpoints.

On the basis of an interim consensus approach, we propose that AFST for *S. brasiliensis* be performed according abovementioned modified CLSI M38 protocol, with the pure mycelial phase at 30°C, confirmed by microscopy and preceded by culture on PDA at 30°C for 7 days (Table 1, Figure 2). AFST of the yeast phase can alternatively be performed but, in short, full transition to the correct phase requires prolonged culture on brain–heart infusion and incubation at 35°C.

**Figure 2.**
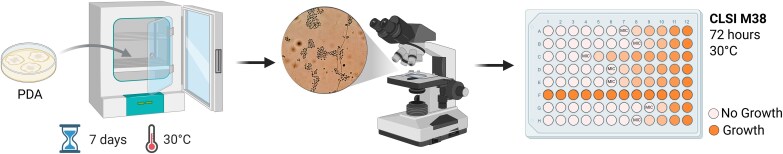
Schematic overview of the proposed AFST protocol for *S. brasiliensis*.

**Table 1.** Overview of proposed methodology for *S. brasiliensis* AFST

Procedure	Value
Preculture	At 30°C 7 days on PDA^a^
Inoculum concentration	0.4–5 × 10^4^ cfu/mL, measurement at 530 nm
Endpoint reading criteria	At 30°C after 72–96 hours^a^
Quality control strain	*Candida parapsilosis* ATCC 22019
	*Candida krusei* ATCC 6258
	*Aspergillus flavus* ATCC 204304
Recommended antifungals agents and concentration ranges	Amphotericin B: 0.016–16 µg/mL
	Itraconazole: 0.016–16 µg/mL
	Terbinafine: 0.004–4 µ g/mL

ATCC, American Type Culture Collection; cfu, colony forming unit.

^a^Deviating from the CLSI M38 standard.
